# Performance of ChatGPT in emergency medicine residency exams in Qatar: A comparative analysis with resident physicians

**DOI:** 10.5339/qmj.2024.61

**Published:** 2024-11-11

**Authors:** Haris Iftikhar, Shahzad Anjum, Zain A. Bhutta, Mavia Najam, Khalid Bashir

**Affiliations:** 1 Emergency Medicine, Hamad General Hospital, Doha, Qatar *Email: haris.ifti@gmail.com; 2 Department of Medical Education, Hamad Medical Corporation, Doha, Qatar

## Abstract

**Introduction:**

The inclusion of artificial intelligence (AI) in the healthcare sector has transformed medical practices by introducing innovative techniques for medical education, diagnosis, and treatment strategies. In medical education, the potential of AI to enhance learning and assessment methods is being increasingly recognized. This study aims to evaluate the performance of OpenAI’s Chat Generative Pre-Trained Transformer (ChatGPT) in emergency medicine (EM) residency examinations in Qatar and compare it with the performance of resident physicians.

**Methods:**

A retrospective descriptive study with a mixed-methods design was conducted in August 2023. EM residents’ examination scores were collected and compared with the performance of ChatGPT on the same examinations. The examinations consisted of multiple-choice questions (MCQs) from the same faculty responsible for Qatari Board EM examinations. ChatGPT’s performance on these examinations was analyzed and compared with residents across various postgraduate years (PGY).

**Results:**

The study included 238 emergency department residents from PGY1 to PGY4 and compared their performances with ChatGPT. ChatGPT scored consistently higher than resident groups in all examination categories. However, a notable decline in passing rates was observed among senior residents, indicating a potential misalignment between examination performance and practical competencies. Another likely reason can be the impact of the COVID-19 pandemic on their learning experience, knowledge acquisition, and consolidation.

**Conclusion:**

ChatGPT demonstrated significant proficiency in the theoretical knowledge of EM, outperforming resident physicians in examination settings. This finding suggests the potential of AI as a supplementary tool in medical education.

## 1. Introduction

The integration of artificial intelligence (AI) in healthcare has revolutionized medical practices, introducing novel approaches to medical education, diagnosis, and treatment strategies. Among the groundbreaking advancements, OpenAI’s Chat Generative Pre-Trained Transformer (ChatGPT) stands out for its remarkable text generation and interpretation capabilities. This study focuses on evaluating the performance of ChatGPT in emergency medicine (EM) residency examinations in Qatar, juxtaposed against resident physicians’ performance.

In the realm of medical education, AI’s potential to enhance learning and assessment methods has been increasingly acknowledged. Studies have shown that AI, especially in the form of large language models like ChatGPT, holds promise in various medical educational contexts.^1,2^ The ability of AI to process and analyze vast amounts of data offers an unparalleled advantage in creating a more dynamic and interactive learning environment. This is particularly relevant in EM, a field that demands quick decision-making and a thorough understanding of diverse medical scenarios.^3^

AI’s role in educational assessment, particularly in the format of multiple-choice questions (MCQs), is a burgeoning field of interest. MCQs are a widely used tool in medical examinations due to their objectivity and ability to cover a broad range of topics efficiently.^4^ The effectiveness of AI in generating and answering MCQs can provide insights into its potential as an educational tool in medicine. Furthermore, the comparison of AI’s performance with that of human trainees offers valuable perspectives on the strengths and limitations of both, and how they can complement each other in medical education.^5,6^

EM, characterized by its fast-paced and high-pressure environment, requires residents to rapidly assimilate a wide range of medical knowledge and apply it effectively. The training programs in Qatar have been designed to equip residents with the necessary skills and knowledge to manage critical cases efficiently.^3^ However, there is a paucity of research on the application of AI in this specific medical field, especially in the Middle Eastern context. This study aims to bridge this gap by analyzing and comparing the performances of ChatGPT and EM residents in residency examinations.

## 2. Methods

This retrospective descriptive study, employing a mixed-methods approach, was initiated following the receipt of ethical approval on August 23, 2023 (MRC 01-23-502) from Hamad Medical Corporation (HMC) Medical Research Centre. We adopted a convenience sampling strategy for our research. The inclusion criteria for the study were straightforward: we incorporated the scores of all EM residents enrolled in the Department of Emergency Medicine Residency Training Program. Conversely, we excluded residents whose scores were unavailable in the program database due to absence from the examination for reasons like annual, casual, or sick leave, ensuring a comprehensive and representative sample of current trainees.

In terms of data collection, we collated anonymized performance data of EM trainees. This data encompassed their examination scores between October 2021 and September 2022, consisting of a total of five examinations (Oct-21, Dec-21, Feb-22, Jun-22, and Sep-22). The examinations in question consisted of single-best-answer MCQs devised by faculty members who were also involved in constructing the Qatari Board EM examinations. Each examination consists of 40 MCQ questions, and every question has four options to choose from. The examinations were conducted using paper and pencil in a designated examination hall, where residents had 60 minutes to complete the examination. All residents from various postgraduate years (PGY) levels took the same examination simultaneously. The topics of each examination were distinct, and each year, new examinations were designed by updating the question bank with evidence-based information from the core faculty and examination committee. For the validation of correct answers in MCQ examinations, a comprehensive approach was taken. EM program directors and core faculty, who are subject matter experts, initially reviewed and aligned questions with the curriculum, followed by rigorous cross-referencing with authoritative medical sources. These questions underwent a thorough peer review and were pilot tested with all residents yearly to assess clarity and difficulty. The peer-reviewed and pilot-tested MCQs are added to the department’s confidential MCQ pool, from which future examinations are made. After the examinations, feedback from participants and educators also facilitated regular updates, ensuring the examination’s reliability in reflecting the evolving field of EM.

In a unique aspect of our study, these examinations were administered to ChatGPT 4.0 (paid version) in May 2023 for performance assessment. During this time, ChatGPT 4.0 did not possess image recognition capabilities; therefore, any images in the examinations were converted into text descriptions for evaluation. MCQs were transcribed into ChatGPT in batches of 10, following which the answers provided by the AI-powered assistant were verified, documented, and evaluated. The performance of ChatGPT was then scored and juxtaposed against the scores of EM residents across different PGY, ranging from PGY1 to PGY4. This comparison, sanctioned by the Institutional Review Board (IRB), aimed to critically evaluate ChatGPT’s proficiency and familiarity with current EM clinical guidelines, as cited in references.^7,8^

For the statistical analysis of our data, we employed STATA software. The analysis included using linear regression to assess associations between category-level scores and examining performance differences utilizing Chi-square tests, Fisher’s exact test, and univariable logistic regression. Throughout this analytical process, we maintained a significance level of P < 0.05. It is important to note that our study meticulously adhered to the strengthening the reporting of observational studies in epidemiology (STROBE) guidelines to ensure the robustness and reliability of our research findings.

## 3. Results

In this study, we evaluated the performances of 238 EM residents from the emergency department, spanning from PGY1 to PGY4, and compared them with the AI language model, ChatGPT, as detailed in Table 1. The participant demographics were diverse, encompassing 58 PGY1 residents (23.8%), 61 PGY2 residents (25.1%), 66 PGY3 residents (27.2%), and 53 PGY4 residents (21.8%), with a gender distribution showing a male-to-female ratio of approximately 2:1. The minimum passing scores for each PGY level were as follows: PGY4: 60%, PGY3: 55%, PGY2: 50%, and PGY1: 45%. Notably, each examination comprised 40 questions, and the maximum score for each was 40.

Focusing on the performance of EM trainees, the results were indicative of a progressive learning curve throughout the residency program. PGY1 residents, at the outset of their training, scored an average of 18 ± 3.5, laying down a foundational knowledge base. This was followed by a slight improvement among PGY2 residents, who achieved an average score of 19.4 ± 3.2. The upward trend continued with PGY3 residents, who demonstrated further progress, scoring an average of 21.1 ± 3.8. The most senior group, the PGY4 residents, attained the highest average score of 21.9 ± 4.2, reflecting their continuous learning and skill development throughout the residency program.

A remarkable aspect of our study was the comparison with ChatGPT. Surprisingly, ChatGPT outperformed all resident groups, scoring an impressive average of 25.8 ± 2.6. Statistical analysis underscored significant differences in performance between the EM trainees and ChatGPT. This difference pointed towards ChatGPT’s advanced proficiency in medical knowledge and problem-solving.^9,10^

An intriguing pattern emerged when analyzing the passing rates trend. Contrary to expectations, 64.7% of PGY1 residents passed the examinations, whereas only 42% of PGY4 residents achieved passing scores.

## 4. Discussion

The findings of this study offer a compelling insight into the capabilities of AI, particularly ChatGPT, in the context of EM education. ChatGPT’s performance, outstripping that of resident physicians across all PGY, marks a significant milestone in the intersection of AI and medical education. This achievement corroborates the growing body of research underscoring the potential of AI as a supplementary tool in various educational settings, including medical training.^1^

The study used a retrospective descriptive methodology with a mixed-methods design, providing a comprehensive analysis of the performances of both ChatGPT and EM residents. By including a wide range of participants across different PGY and comparing their performances on standardized examinations, the study offers a robust evaluation of ChatGPT’s capabilities in the context of EM. This approach aligns with the current trends in medical education research, which emphasize the need for innovative methods to enhance learning and assessment processes.^10,11^

The consistent improvement in scores across the EM training years highlights the efficacy of the existing residency programs in Qatar in enhancing medical knowledge and clinical decision-making skills.^3^ However, the performance of ChatGPT indicates the added value that AI can bring to medical education, especially in standard theoretical knowledge and problem-solving abilities. This observation is in line with studies demonstrating AI’s proficiency in processing vast amounts of data and applying knowledge in diverse scenarios.^1,12^

The decline in passing rates among senior residents raises questions about the alignment between examination formats and the practical competencies developed throughout the residency. This suggests a potential gap in the assessment methods used in EM training, where theoretical knowledge may not entirely capture the complexities of real-life medical scenarios. This finding echoes the sentiments of recent literature advocating for a more holistic approach in medical education, emphasizing the integration of practical skills and humanistic qualities alongside theoretical knowledge.^5,13^ Another likely reason can be the impact of the COVID-19 pandemic on their learning experience, knowledge acquisition, and consolidation. To effectively prepare for future pandemics, we must prioritize the safety and security of our teaching environment while ensuring residents’ well-being. This can only be achieved through rigorous safeguards, including adequate support in medical education and supervision.^14,15^

Recently, ChatGPT demonstrated remarkable success in medical licensing and other examinations and continues to improve. ChatGPT achieved a passing score for a third-year medical student by surpassing the 60% threshold on the NBME-Free Step-1 dataset.^6^ GPT-4 surpassed GPT-3 and successfully passed all six years of the Japanese medical examinations, demonstrating its potential in a different language.^9^ ChatGPT performed well on the European Examination in Core Cardiology (EECC) multiple choice questions, consistently scoring above 60%, which was usually the pass mark for the candidates.^16^ In the European Board Examination of the Nuclear Medicine Section, ChatGPT was put to the test with fifty MCQs. ChatGPT provided a definitive answer 34% of the time. However, the mean probability of randomly selecting the correct answer was only 0.24, indicating that ChatGPT possesses some knowledge rather than simply guessed.^1,8^

Completing medical registration in Germany involves passing three state examinations. The first examination (M1) covers pre-clinical subjects, while the second examination (M2) is a written test that assesses medical specialties. ChatGPT passed German medical examinations M1 and M2 with 60.1% and 66.7% with a passing grade on each exam.^8^

In another study of 50 questions from EM, human test-takers achieved a mean correct response rate of 83.7%, outperforming GPT-3.5 (61.4%) but not GPT-4 (82.1%). The study found a statistically significant difference in performance between GPT-3.5 and both human beings and GPT-4 (mean difference: 21.5%), while no statistically significant difference was observed between human performance and GPT-4 (mean difference: 1.6%).^17^

A study on the bar examination found that GPT-4 can pass the uniform bar examination without prior training, challenging the assumption that domain-specific models would struggle. With a deep understanding of legal concepts and excellent reading and writing skills, large language models like GPT-4 can meet the same standard as human lawyers.^10^

The study’s results underscore the need for further research into the application of AI in medical education, particularly in specialties like EM that require a unique blend of knowledge, skills, and decision-making abilities. While ChatGPT has shown proficiency in theoretical knowledge, its current limitation in image analysis, a crucial aspect of EM diagnostics, points towards areas needing improvement. Future iterations of AI tools capable of interpreting clinical images could significantly enhance their utility in medical education and practice.^3,18^

Moreover, the study’s findings highlight the dynamic nature of AI development and the challenges it poses in establishing a consistent assessment framework. As AI tools rapidly evolve, keeping pace with their capabilities and integrating them effectively into medical education becomes imperative. This aligns with recent discussions on the role of AI in reshaping educational paradigms, advocating for adaptive and flexible teaching and assessment strategies that can accommodate the rapid advancements in technology.^19,20^

ChatGPT achieved an impressive average score of 25.8 ± 2.6 out of 40. However, it is still far from perfect considering its percentage out of the total. Even though advanced, ChatGPT still does not fully grasp the complexities of medical scenarios, where decision-making often involves synthesizing information from diverse sources.^21^ There is a concern that AI and ChatGPT may inadvertently reinforce cognitive biases in its training data, leading to potential errors in clinical reasoning and decision-making.^22^ It is unclear whether AI models can process real-time patient data or adjust their recommendations dynamically during ongoing care.^23^ Additionally, AI might lack the ability to make ethical judgments, which is often required in EM, such as in cases involving triage, end-of-life care, or complex consent issues.^24^ There is a risk that AI models might generate recommendations that are not evidence-based or that contradict established medical guidelines, potentially leading to harmful outcomes if relied upon inappropriately.^25^ While ChatGPT has the potential for benefits in EM education, it is important to be cautious as it also has limitations in clinical training and practice.

The study not only demonstrates ChatGPT’s formidable capabilities in understanding and applying medical knowledge but also suggests a potential transformative role for AI in medical education and healthcare in general. The integration of AI, with its unique strengths and evolving capabilities, alongside traditional training methods, could significantly enhance the learning experience and the overall quality of medical education. However, the art of medicine, with its inherent humanistic qualities and practical skills, remains a domain that AI cannot replace. The complementary use of AI and human expertise could be the key to optimizing medical education and improving healthcare outcomes.^11,17,26^

We want to acknowledge that there are several limitations in our study. The study was conducted in a single facility and program, which may limit the generalizability of the findings. Future research should aim for a more diverse sample to ensure broader applicability and relevance across various healthcare settings. This study only included MCQs, and we cannot compare the performance of ChatGPT on other types of questions, such as short-answer questions (SAQ) or objective structured clinical examinations (OSCEs), which are also commonly used in international medical examinations.^27-29^ Furthermore, at the time of the study, ChatGPT did not have image recognition capabilities, so we could not assess its image recognition abilities and knowledge. Lastly, we should also consider that the potential impact of the pandemic may have affected different groups of residents at various stages of their medical education, which may have affected their scores and influenced ChatGPT’s performance comparison.

## 5. Conclusion

The study analyzed the performance of EM trainees during their residency program and found that their knowledge and scores improved as they progressed through the program. However, an AI-powered language model, ChatGPT, outperformed all the resident groups in medical knowledge and clinical problem-solving.

In summary, this study not only contributes to the emerging body of literature on AI in medical education but also provides practical insights that could shape future training and assessment strategies in EM. As AI continues to evolve and integrate into various aspects of healthcare, understanding its potential and limitations becomes crucial for optimizing its benefits in medical education and practice.

In light of these findings, future research should focus on exploring the practical applications of AI in various medical specialties, assessing its impact on both theoretical and practical competencies, and developing strategies to integrate AI effectively into medical training programs. The ultimate goal is to harness the full potential of AI in enhancing the quality of medical education and healthcare delivery, thereby benefiting patients and healthcare providers alike.

## Declaration of Interests

The authors declare that they have no known competing financial interests or personal relationships that could have appeared to influence the work reported in this paper.

## Acknowledgment

NA.

## Authorship Declaration

All authors agree with the content of the manuscript.

## MRC Approval

Ethical approval was obtained from the Hamad Medical Corporation’s Institutional Review Board (MRC 01-23-502).

## Authors’ Contributions

HI, SA, MN, KB: Conceptualization; Writing—original draft preparation; Data curation; Funding acquisition; Methodology; Project administration. ZB: Formal analysis; Validation; Writing—review and editing.

## Figures and Tables

**Table 1.** Comparison of marks obtained and percentages for trainees and ChatGPT for examination sessions.

**Variable**	**Category**	**PGY1 *N* = 58**	**PGY2 *N* = 61**	**PGY3 *N* = 66**	**PGY4 *N* = 53**	**GPT *N* = 5**	**Total**	***p*-value**
Gender, *n* (%)	Male	28 (48.3)	43 (70.5)	55 (83.3)	39 (73.5)		165 (69.3)	
	Female	30 (51.7)	18 (29.5)	11 (16.6)	14 (26.4)		73 (30.6)	
Obtained mean examination score, Mean ± SD	Total	18 ± 3.5	19.4 ± 3.2	21.1 ± 3.8	21.9 ± 4.2	25.8 ± 2.6	20.4 ± 3.9	< 0.05
	Oct-21	14.4 ± 2.9	17.1 ± 2.5	21.3 ± 2.3	22.7 ± 3.7	23	19.1 ± 4.2	
	Dec-21	20.8 ± 1.9	21.3 ± 3.4	21.9 ± 3.1	23.8 ± 2.1	26	21.9 ± 2.7	
	Feb-22	20.8 ± 2.8	22.3 ± 2.8	24.7 ± 4.1	25.9 ± 2.4	25	23.4 ± 3.6	
	Jun-22	17.9 ± 1.8	18.3 ± 2.8	19.2 ± 2.1	18.6 ± 4.2	25	18.6 ± 2.8	
	Sep-22	17.5 ± 2.2	18.3 ± 2.5	17.3 ± 3	18.5 ± 3.4	30	18.2 ± 3.3	
								
Percentage mean examination score, Mean ± SD	Total	46.4 ± 8.4	48.6 ± 8.1	52.8 ± 9.6	54.7 ± 10.6	65.5 ± 6.5	51.0 ± 9.9	< 0.05
	Oct-21	36.3 ± 7.2	42.7 ± 6.3	53.2 ± 5.7	56.7 ± 9.1	57.5	47.7 ± 10.4	
	Dec-21	52.1 ± 4.8	53.3 ± 5.9	54.8 ± 7.8	59.5 ± 5.2	65	54.9 ± 6.7	
	Feb-22	52.1 ± 7.1	55.6 ± 7.2	61.7 ± 10.1	64.7 ± 5.9	67.5	58.6 ± 9.1	
	Jun-22	44.7 ± 4.5	45.7 ± 7.2	47.9 ± 5.1	46.5 ± 10.4	62.5	46.6 ± 7.1	
	Sep-22	43.7 ± 5.4	45.7 ± 6.2	43.2 ± 7.5	46.2 ± 8.6	75	45.6 ± 8.4	
Status, *n* (%)	Pass	33 (64.7)	28 (47.5)	26 (40.6)	21 (42)		108 (48.2)	
	Fail	18 (35.3)	31 (52.5)	38 (59.4)	29 (58)		116 (51.7)	
	Absent	7 (12.1)	2 (3.3)	2 (3.0)	3 (5.6)			
Pass, *n* (% out of passed candidates)	Oct-21	2 (6.1)	2 (7.1)	6 (23.1)	4 (19.1)		14 (30.4)	
	Dec-21	12 (36.3)	8 (28.6)	8 (30.7)	6 (28.6)		34 (70.8)	
	Feb-22	10 (30.3)	9 (32.1)	10 (38.5)	9 (42.3)		38 (79.2)	
	Jun-22	7 (21.2)	5 (17.8)	2 (7.7)			14 (35)	
	Sep-22	2 (6.1)	4 (14.3)		2 (9.5)		8 (19.1)	

PGY: postgraduate years; *n*: Number; SD: standard deviation.
